# The non-high-density lipoprotein cholesterol to high-density lipoprotein cholesterol ratio (NHHR) as predictors of hypertensive patients: Analyses of NHANES data with machine learning

**DOI:** 10.1097/MD.0000000000045829

**Published:** 2025-11-14

**Authors:** Zhaoxing Cao, Fangfang Zhuo, Fei Yan, Runze Huang, Zhangrong Chen

**Affiliations:** a Department of Cardiovascular Medicine, The Affiliated Hospital of Guizhou Medica University, Guiyang, China; b The Affiliated Hospital of Guizhou Medical University, The Key Laboratory of Myocardial Remodeling Research, Guiyang, China.

**Keywords:** Boruta algorithm, hypertension, machine learning, NHANES, NHHR, restricted cubic spline model

## Abstract

Elevated values of the non-HDL/HDL cholesterol ratio (NHHR) have been associated with increased hypertension risk, indicating its potential as a pathogenic factor, but its assessment remains challenging. We analyzed data from 22,562 hypertensive participants in the National Health and Nutrition Examination Survey (NHANES) 2009 to 2018, employing predictive algorithms to evaluate the NHHR index’s ability to forecast hypertension outcomes. We found that the risk of hypertension was higher in the highest than in the lowest NHHR tertile. Weighted logistic regression showed revealed a statistically significant positive correlation of NHHR with hypertension prevalence in the fully adjusted model. Restricted cubic-spline analysis showed a linear association in the fully adjusted model. Subgroup analysis indicated that significant interactions between NHHR and hypertension were observed in the subgroups of race, smoking, and educational level. Boruta, algorithm corroborated that NHHR is an important predictor of hypertension. Among the 8 machine-learning models evaluated for predictive capabilities, CatBoost methods are used to construct the models, and their performance is evaluated, with an area under the curve of 0.804. Therefore, NHHR is a significant predictor of hypertensive patients. Incorporating these factors into risk prediction algorithms enhances classification accuracy and facilitates earlier detection of at-risk subjects in this cohort.

## 1. Introduction

Hypertension is a common chronic uninfectious disease that affects the health of people worldwide and causes 10.4 million deaths each year.^[1]^ The 3-decade period from 1990 through 2019 saw global hypertension prevalence surge from 650 million to 1.3 billion patients.^[2]^ Well-established as a critical contributor to stroke, cardiovascular disorders, and renal impairment.^[3–5]^ Globally, hypertension contributes to an alarming 8.5 million annual deaths.^[6]^ The high prevalence and mortality rates of hypertension result in significant physical harm to patients and impose a heavy burden on society and families.^[2,7]^ Timely detection and management of hypertension in at-risk populations can mitigate complications while representing an economically viable care approach.

Non-high-density lipoprotein cholesterol (non-HDL-C) is considered a pivotal contributor to cardiovascular (CV) disease.^[8]^ Two important risk factors for atherosclerotic CV disease are hypertension and dyslipidemia.^[9]^ Emerging evidence demonstrates that obesity and dyslipidemia are interrelated metabolic risk factors contributing to the development of hypertension.^[10]^ The non-high-density lipoprotein (NHDL) cholesterol to HDL-C ratio (NHHR) is an emerging comprehensive indicator of atherosclerotic lipid.^[11]^

While NHHR has been linked to hypertension pathogenesis, research examining this connection is still scarce. Additionally, the application of computational modeling approaches to assess NHHR-hypertension correlations warrants further investigation. In this study, we hypothesized that Higher NHHR values are independently linked to greater hypertension susceptibility. We utilized various analytic approaches, including weighted logistic regression, restricted cubic spline (RCS), subgroup analysis, random forest analysis, Boruta algorithm, and 8 machine-learning models to confirm the link between them, with the objective of refining risk classification and facilitating adverse event prevention strategies.

## 2. Methods

### 2.1. Study population and design

All data and guidance on analytical approaches are publicly and freely available from the US Centers for Disease Control and Prevention’s National Center for Health Statistics and can be assessed at https://www.cdc.gov/nchs/nhanes/index.htm. The study protocols were approved by the NCHS Research Ethics Review Board, and all participants provided written informed consent. This study was reported according to the Strengthening the Reporting of Observational Studies in Epidemiology (STROBE) guidelines.^[12]^ The current study did not require additional ethical approval or informed consent owing to its nature as a secondary data analysis.

This cross-sectional analysis used 10 years of data from the 2009 to 2018 National Health and Nutrition Examination Survey (NHANES) cycles. The total combined sample of NHANES 2009 through 2018 (the most recent complete data given the disruptions caused by the COVID-19 pandemic) comprised 49,693 participants. First, we excluded participants with missing data for the hypertension variable, including cases with “Don’t know” or “Refused” responses (15,743 individuals). Second, we excluded individuals with missing data for the NHHR variable (3169 individuals). Lastly, we excluded participants with incomplete data on any covariates (6241 individuals). A total of 22,562 eligible participants were included.

### 2.2. Assessment of NHHR

The NHHR was calculated based on serum total cholesterol and HDL-C levels from the NHANES database for the years 1999 to 2018. Non-HDL-C is defined as total cholesterol minus HDL-C, and the NHHR is calculated as the ratio of non-HDL-C to HDL-C.^[13]^ Participants were divided into 4 groups (Q1, Q2, Q3, and Q4) based on the NHHR quartiles, with the Q1 group serving as the reference group.

### 2.3. Definition of hypertension

Experienced examiners in the NHANES group recorded the blood pressure of the participants according to the guideline of the American Heart Association.^[14]^ Hypertension was defined as follows: average systolic blood pressure 140 mm Hg, average diastolic blood pressure 90 mm Hg, self-reported hypertension, individuals with prescribed antihypertensive medications. Meeting any one of these criteria signifies the presence of hypertension.^[15]^

### 2.4. Covariates

We acquired demographic information such as age, sex, race, and education level from the demographic questionnaire. The sociodemographic characteristics included age, sex, race, education level, marital status, and the PIR. For lifestyle behaviors, participants who smoked over 100 cigarettes throughout their lifetime were defined as smokers, regardless of whether he/she had quitted smoking at the time of interview,^[16]^ and those consuming at least 12 drinks during the year preceding the survey were considered alcohol drinkers.^[17]^ Health factors consisted of BMI (normal, overweight or obese) and a history of angina pectoris, diabetes, and cancer based on the self-reported questionnaire.

### 2.5. Statistical analysis

DecisionLinnc1.0 software was employed for data analysis.^[18]^ DecisionLinnc1.0 is a platform that integrates multiple programming language environments and enables data processing, data analysis, and machine learning through a visual interface. The data were weighted according to the standard sample weighting guidelines of the NHANES to ensure sample representativeness. We first compared the sample characteristics between participants with and without hypertension using the Chi-square test for categorical variables and the Wilcoxon rank-sum test for continuous variables. Subsequently, we employed a multivariable logistic regression model to investigate the association between NHHR (as a continuous variable) and hypertension, using odds ratios and 95% confidence intervals (CI).

Three Cox models were constructed with different levels of adjustment: Model 1 was unadjusted; Model 2 was adjusted for age, race, sex, education level, PIR, and marital status; Model 3 was further adjusted for BMI, smoking, diabetes, angina pectoris, total cholesterol, and cancer. RCS analysis was performed to assess nonlinear relationships of the NHHR and risk of angina pectoris. Additionally, the Boruta algorithm was used to assess variable importance.

The following machine-learning algorithms were applied to the dataset after variable selection: Naive Bayes, logistic regression, random forest, multi-layer perceptron, boosted trees, support vector machine with radial basis function kernel, decision tree, and K-nearest neighbors. In our cases, the model with the highest area under the curve (AUC) of the receiver operating characteristic (ROC) curve was selected as the best model for each algorithm. To assess the robustness of the study results across different sample characteristics, we performed subgroup analyses. A two-sided *P*-value of* < *0.05 was considered statistically significant. Variance inflation factor (VIF) values were calculated to test for multicollinearity, with a VIF *< *5 indicating no multicollinearity.

## 3. Results

### 3.1. Baseline characteristics of the participants

Table 1 shows that a total of 22,562 participants were included in the study. 7996 reported a history of hypertension (35.44%). The mean age was 49.03 ± 17.59 years, with 51,70% women (n = 11,664). The average NHHR was 2.89 ± 1.44. Significant differences (*P < *.05) were observed across demographic (age, race, marital status), socioeconomic (education, PIR), and clinical characteristics (BMI, smoking status, and prevalence of angina pectoris, diabetes, and cancer). Notably, hypertensive individuals showed significantly higher NHHR than non-hypertensive subjects (2.95 ± 1.43 vs 2.86 ± 1.44 mg/dL, *P < *.001).

**Table 1:** Sample characteristics and comparison between participants with and without hypertension (unweighted).

Characteristic	Overall	No hypertension	Hypertension	*P*-value
	N = 22,562	N = 14,566	N = 7,996	
Sex				
Female	11,664.00 (51.70%)	7544.00 (51.79%)	4120.00 (51.53%)	.702
Male	10,898.00 (48.30%)	7022.00 (48.21%)	3876.00 (48.47%)	
Race				
Mexican American	3193.00 (14.15%)	2290.00 (15.72%)	903.00 (11.29%)	*<*.001
Non-Hispanic Black	4637.00 (20.55%)	2541.00 (17.44%)	2096.00 (26.21%)	
Non-Hispanic White	9376.00 (41.56%)	5941.00 (40.79%)	3435.00 (42.96%)	
Other Hispanic	2247.00 (9.96%)	1518.00 (10.42%)	729.00 (9.12%)	
Other Race	3109.00 (13.78%)	2276.00 (15.63%)	833.00 (10.42%)	
Education level				
<9th grade	2096.00 (9.29%)	1217.00 (8.36%)	879.00 (10.99%)	*<*.001
9–11th grade	2905.00 (12.88%)	1772.00 (12.27%)	1133.00 (14.17%)	
Some college or AA degree	6961.00 (30.85%)	4476.00 (30.73%)	2485.00 (31.08%)	
College, graduate or above	5529.00 (24.51%)	3994.00 (27.42%)	1535.00 (19.20%)	
Marital status				
Divorced	2486.00 (11.02%)	1365.00 (9.37%)	1121.00 (14.02%)	*<*.001
Living with partner	1882.00 (8.34%)	1430.00 (9.82%)	452.00 (5.65%)	
Married	11,564.00 (51.25%)	7385.00 (50.70%)	4179.00 (52.26%)	
Never married	4201.00 (18.62%)	3362.00 (23.08%)	839.00 (10.49%)	
Separated	751.00 (3.33%)	460.00 (3.16%)	291.00 (3.64%)	
Widowed	1678.00 (7.44%)	564.00 (3.87%)	1114.00 (13.93%)	
Smoking				
No	12,723.00 (56.39%)	8702.00 (59.74%)	4021.00 (50.29%)	*<*.001
Yes	9839.00 (43.61%)	5864.00 (40.26%)	3975.00 (49.71%)	
Age	49.03 ± 17.59	43.28 ± 16.24	59.51 ± 14.91	*<*.001
PIR	2.48 ± 1.63	2.51 ± 1.65	2.42 ± 1.59	*<*.001
BMI	29.28 ± 7.08	28.15 ± 6.61	31.33 ± 7.44	*<*.001
Total Cholesterol	191.41 ± 41.71	192.07 ± 40.66	190.21 ± 43.53	.002
HDL C	53.16 ± 16.19	53.79 ± 16.13	52.02 ± 16.22	*<*.001
NHHR	2.89 ± 1.44	2.86 ± 1.44	2.95 ± 1.43	*<*.001
Angina pectoris				*<*.001
No	22,015.00 (97.58%)	14,449.00 (99.20%)	7566.00 (94.62%)	
Yes	547.00 (2.42%)	117.00 (0.80%)	430.00 (5.38%)	
Diabetes				
No	19,515.00 (86.49%)	13,643.00 (93.66%)	5872.00 (73.44%)	*<*.001
Yes	3047.00 (13.51%)	923.00 (6.34%)	2124.00 (26.56%)	
Cancer				
No	20,464.00 (90.70%)	13,681.00 (93.92%)	6783.00 (84.83%)	*<*.001
Yes	2098.00 (9.30%)	885.00 (6.08%)	1213.00 (15.17%)	

Characteristics of the study participants (n = 23,414).

AA = Associate of Arts, BMI = body mass index, NHHR = non-HDL-C to HDL-C ratio, PIR = poverty-to-income ratio.

### 3.2. Association between NHHR and hypertension

Table 2 shows the association between NHHR and hypertension across all 3 models, using multiple logistic regression models. A significant association was observed between NHHR (treated continuously) and hypertension prevalence in models with and without covariate adjustment. After comprehensive adjustment in Model 3, every 1-unit increment in NHHR corresponded to an 8% elevated hypertension risk. Upon quartile stratification, Q2 demonstrated significant positive associations with all-cause mortality in both crude and partially adjusted models (Model 2), though these effects were attenuated upon full adjustment. The observed VIF range (1.04–1.30) for all included variables demonstrated no evidence of collinearity in our regression models.

**Table 2:** Association between the NHHR and hypertension by multiple logistic models (weighted).

Exposure	Model 1 OR (95% CI)	*P*-value	Model 2 OR (95% CI)	*P*-value	Model 3 OR (95% CI)	*P*-value
NHHR	1.10 (1.07,1.13)	*<*.001	1.13 (1.10,1.17)	*<*.001	1.08 (1.04,1.12)	*<*.001
Categories						
Q 1	Reference		Reference		Reference	
Q 2	1.22 (1.08,1.38)	.002	1.27 (1.11,1.46)	.001	1.08 (0.94,1.24)	.298
Q 3	1.43 (1.27,1.62)	*<*.001	1.59 (1.39,1.82)	*<*.001	1.23 (1.06,1.42)	.007
Q 4	1.56 (1.35,1.79)	*<*.001	1.92 (1.64,2.25)	*<*.001	1.38 (1.14,1.66)	.001

Model 1: Unadjusted model; Model 2: Adjusted for age, sex, race, education level, marital status, and the poverty-to-income ratio; Model 3: Additionally adjusted for BMI, smoking, angina pectoris, diabetes, total cholesterol, and cancer.

CI = confidence interval, NHHR = non-HDL-C to HDL-C ratio, OR = odds ratio.

### 3.3. Subgroup analysis

Stratified analyses by demographic and clinical characteristics (sex, race, marital status, education, smoking status, and comorbidity profiles) revealed consistent associations between NHHR and hypertension, as detailed in Table 3. We observed significant interactions between NHHR and hypertension in the race, smoking and educational level subgroups (*P < *.05).

**Table 3:** Subgroup analysis of the association between NHHR and hypertension.

Characteristic	OR (95% CI)	*P*-value	*P* for interaction
Diabetes			
No	1.11 (1.06, 1.15)	*<*.001	.86
Yes	1.03 (0.92, 1.14)	.636	
Angina pectoris			
No	1.12 (1.08, 1.16)	*<*.001	.726
Yes	1.08 (0.81, 1.43)	.611	
Smoking			
No	1.19 (1.13, 1.25)	*<*.001	*<*.001
Yes	1.06 (1.01, 1.12)	.031	
Cancer			
No	1.13 (1.08, 1.17)	*<*.001	.4
Yes	1.11 (1.01, 1.22)	.033	
Sex			
Female	1.16 (1.10, 1.24)	*<*.001	.448
Male	1.06 (1.01, 1.12)	.027	
Race			
Mexican American	0.99 (0.90, 1.09)	.849	.017
Non-Hispanic Black	1.06 (0.99, 1.14)	.102	
Non-Hispanic White	1.18 (1.12, 1.24)	*<*.001	
Other Hispanic	1.15 (1.03, 1.28)	.019	
Other Race	1.06 (0.95, 1.18)	.276	
Educational level			
9–11th grade	0.97 (0.90, 1.05)	.51	.021
College, graduate or above	1.27 (1.17, 1.39)	*<*.001	
High school graduate	1.07 (0.99, 1.15)	.091	
<9th grade	1.04 (0.92, 1.17)	.512	
Some college or AA degree	1.11 (1.03, 1.19)	.008	
Marital status			
Divorced	1.05 (0.95, 1.16)	.375	.476
Living with partner	1.11 (1.00, 1.24)	.057	
Married	1.16 (1.11, 1.22)	*<*.001	
Never married	1.12 (1.02, 1.22)	.019	
Separated	1.02 (0.81, 1.30)	.848	
Widowed	1.07 (0.94, 1.23)	.307	

AA = Associate of Arts, CI = confidence interval, NHHR = non-high-density to high-density lipoprotein cholesterol ratio, OR = odds ratio.

### 3.4. RCS analysis

**Figure 1.**
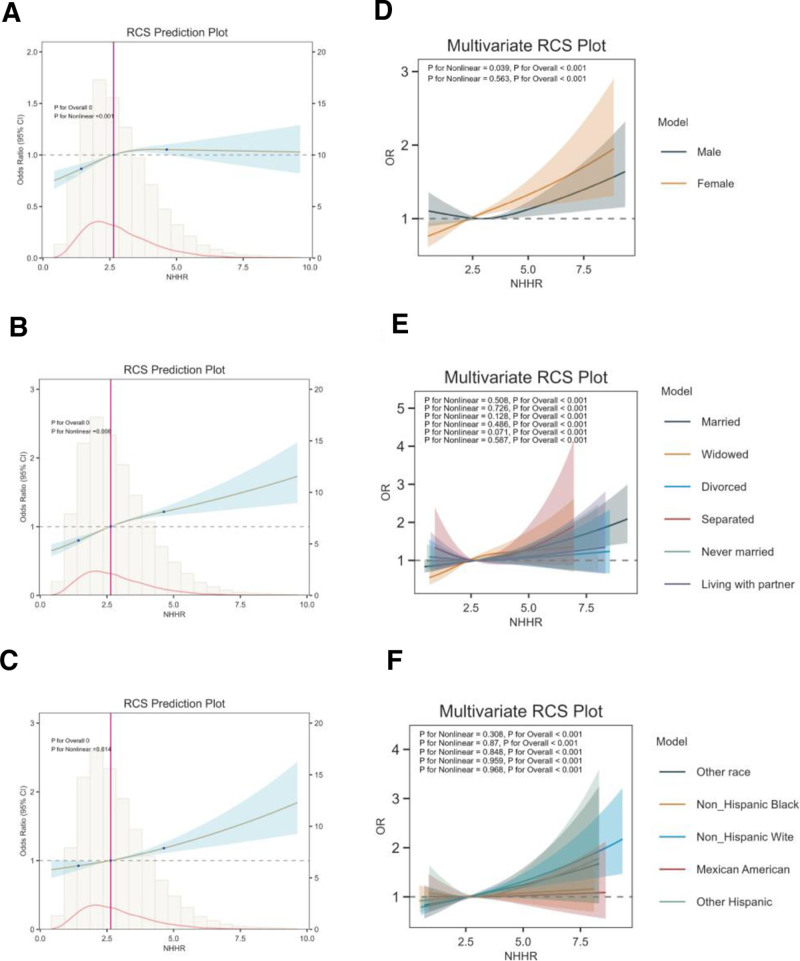
Restricted cubic spline curve (RCS) for the association between NHHR and HTN in model 1 (unadjusted) (A), in model 2 (adjusted for age, sex, race, marital status, education levels, poverty-to-income ratio) (B), and in model 3 (adjusted for age, sex, race, marital status, education levels, poverty-to-income ratio, BMI, TC, smoking, angina pectoris, diabetes and cancer) (C). RCS curve for the association between NHHR and HTN in different sexes (D), in different marital statuses (E), and for different races (F). Lines represent odds ratios (OR), and colored areas represent 95% confidence intervals (CI). BMI = body mass index, HDL-C = high-density lipoprotein cholesterol, HTN = hypertension, NHHR = non-HDL-C to HDL-C ratio, RCS = restricted cubic spline, TC = total cholesterol.

Figure 1A–C shows the RCS curves to assess potential nonlinearity in the relationship between the NHHR and hypertension, with and without controlling for covariates. Nonlinear regression analyses revealed a statistically significant curvilinear relationship between NHHR and hypertension, with *P*-values for nonlinearity reaching .001 in the crude model (Model 1) and .006 in the partially adjusted model (Model 2). In the fully adjusted model (Model 3), the NHHR-hypertension association demonstrated linear characteristics (nonlinearity *P* = .614), supporting a dose-response pattern. Notably, this linear association persisted across population strata defined by sex, race, and marital status (Fig. 1D–F).

### 3.5. Feature selection

**Figure 2.**
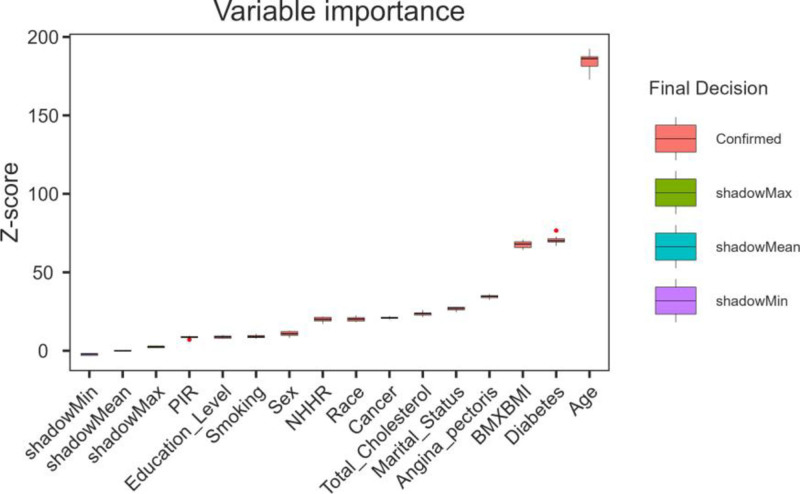
Predictor importance for hypertension according to the Boruta algorithm. BMI = body mass index, HDL-C = high-density lipoprotein cholesterol, NHHR = non-HDL-C to HDL-C ratio, PIR = poverty-to-income ratio.

The feature selection results based on the Boruta algorithm are shown in Figure 2. The 10 variables most strongly associated with hypertension, in order of z-value, were age, diabetes, body mass index (BMI), angina pectoris, marital status, total cholesterol, cancer, race, NHHR, and sex.

Although smoking, educational level and PIR were not the 10 variables most strongly associated with hypertension, they were still included in subsequent analysis based on previous research and clinical experience.

### 3.6. Performance of the 8 machine-learning models

**Figure 3.**
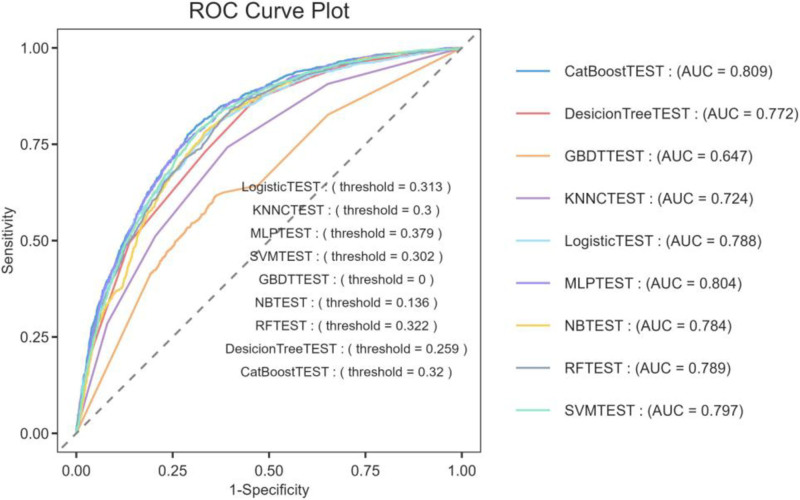
Receiver operating characteristic (ROC) curves of the 8 machine-learning models. AUC Area under the receiver operating characteristic curve.

We evaluated the predictive capabilities of 8 machine-learning models by plotting ROC curves and calculating AUC values. As shown in Figure 3, the CatBoost model had the highest AUC value, with an accuracy of 74.31%, a precision of 66.57%, a recall of 58.19%, and an F1-Score of 62.10%.

**Figure 4.**
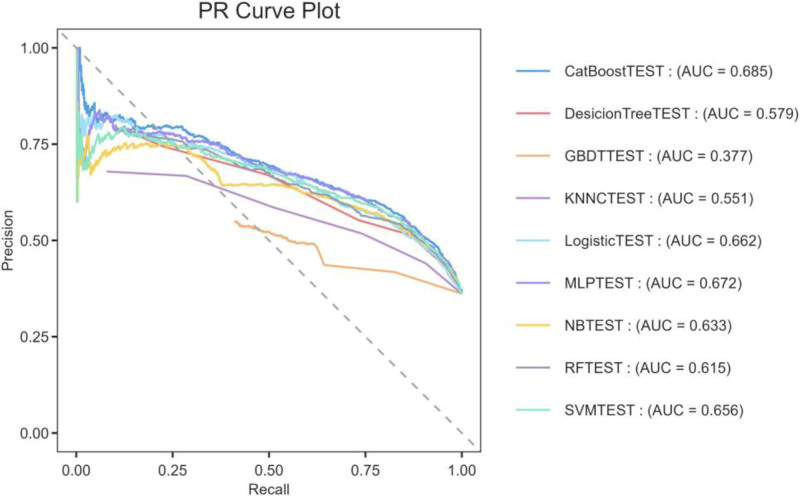
The precision-recall curve for the 8 machine-learning models. AUC = area under curve of the receiver operating characteristic curve.

To overcome class imbalance limitations inherent in ROC analysis, supplementary PR curve evaluation was conducted. CatBoost models consistently outperformed alternatives, as quantified by higher average precision measures (Fig. 4).

## 4. Discussion

To the best of our understanding, this investigation represents the inaugural application of machine learning combined with multidimensional analytical frameworks to examine the NHHR-hypertension relationship utilizing nationally representative survey data. The principal discoveries emerging from our investigation include: Our analyses consistently identified NHHR as a significant positive predictor of hypertension, with this relationship proving robust to variations in both statistical modeling and analytical approach. The significant interactions between NHHR and hypertension in the race, smoking and educational level subgroups were observed (*P < *.05). Fully adjusted RCS modeling identified a linear NHHR-hypertension association, with consistent effects observed across all subgroup categories. According to the Boruta algorithm, this study highlighted the potential value of NHHR in the diagnosis of hypertension. Through systematic comparison of 8 distinct machine-learning algorithms, we gained deeper understanding of NHHR’s predictive value in hypertension risk assessment. Among these models, the CatBoost model showed the highest AUC value and demonstrated superior performance in predicting the occurrence of hypertension, with remarkable accuracy, precision, recall, and F1-Score metrics. Therefore, our research findings indicate that NHHR is a risk factor for hypertension, and its risk prediction has potential clinical applications, which can aid in guiding further prevention and intervention efforts.

The NHHR (non-HDL-C/HDL-C ratio) represents a novel integrated metric of atherogenic lipid profiles, quantifying the equilibrium between pro-atherogenic (non-HDL-C) and anti-atherogenic (HDL-C) lipoprotein fractions. This biomarker has been clinically associated with dyslipidemia-related pathologies^[19]^ that promote vascular structural changes and subsequent blood pressure elevation.^[20]^ Multivariable analyses support an independent association between elevated NHHR levels and incident hypertension risk. An early study suggested that the TyG index and related biomarkers serve as autonomous prognostic indicators for overall and cardiovascular mortality among hypertensive individuals.^[21]^ Jian et al^[22]^ also confirmed an independent positive relationship between the TyG index and incident hypertension. Longitudinal data from the UK Biobank cohort revealed a significant mortality risk associated with extremely elevated HDL-C levels specifically among hypertensive participants.^[23]^ Existing studies have utilized NHANES data from 2001 to 2018 to investigate the association between NHHR and hypertension, revealing a positive correlation between these 2 variables.^[24]^ Moreover, existing studies have highlighted the predictive value of machine learning in cardiovascular diseases.^[25,26]^

Previous studies have proposed several hypotheses regarding the mechanistic link of NHHR as a lipid biomarker for predicting hypertension. First, Atherosclerosis resulting from lipid abnormalities can cause structural changes in large arteries, resulting in a reduction in elasticity.^[27]^ Second, lipid-mediated impairment of the renal microvasculature could manifest as hypertension.^[28]^ Furthermore, plasma lipids enhance 1-adrenergic receptor pressure sensitivity, impair baroreflex function, and are linked to elevated blood pressure.^[29]^

Machine learning, as a branch of artificial intelligence, is becoming increasingly important in the medical field due to its ability to handle large, complex and diverse data.^[30]^ In contrast to conventional analytical approaches that typically presume linear relationships, artificial intelligence algorithms demonstrate superior capability in detecting complex interrelationships between metabolic parameters, sociodemographic variables, and mortality endpoints.^[31]^ The current investigation identified CatBoost as exhibiting enhanced predictive capability relative to competing models. Embedding machine intelligence systems within healthcare decision support infrastructures,^[32]^ Physicians can obtain enhanced risk assessment precision and customize therapeutic approaches for hypertensive patients with elevated risk profiles.^[33]^

Some study limitations merit discussion. First, the retrospective design of NHANES data prevents causal determinations. Notwithstanding multivariable adjustments, residual confounding variables might still influence the results. Second, due to the inherent limitations of cross-sectional analyses, our study cannot definitively establish causal directionality between NHHR and hypertension, as potential reverse causation cannot be excluded. Prospective cohort studies are warranted to elucidate the temporal relationship. Third, Although machine-learning models demonstrate high predictive accuracy, they require external verification across diverse populations to confirm their reliability and clinical utility. Finally, our subgroup analysis was limited to specific sample characteristics and may not have accounted for other potential confounding factors. Future research should include a broader range of subgroup analyses.

## 5. Conclusion

In conclusion, our study identifies the NHHR as a novel and independent risk factor for hypertension, underscoring its role in linking lipid metabolism dysregulation to disease pathogenesis. These findings support the potential integration of NHHR into clinical risk stratification tools, offering a pragmatic approach to identifying high-risk hypertensive individuals. Further studies are warranted to explore the mechanistic pathways underlying this association and to evaluate the generalizability of NHHR across diverse populations. Future efforts should also prioritize translational research to bridge the gap between biomarker discovery and real-world clinical implementation, ultimately improving targeted interventions for hypertension management.

## Acknowledgments

We would like to express our sincere gratitude to all the members of the research team and all the participants who took part in this study.

## Author contributions

**Conceptualization:** Zhaoxing Cao, Runze Huang, Zhangrong Chen.

**Data curation:** Zhaoxing Cao, Fangfang Zhuo, Fei Yan.

**Investigation:** Fangfang Zhuo.

**Methodology:** Fangfang Zhuo, Runze Huang, Zhangrong Chen.

**Project administration:** Zhaoxing Cao, Fangfang Zhuo, Runze Huang, Zhangrong Chen.

**Resources:** Fei Yan.

**Software:** Zhaoxing Cao, Fangfang Zhuo.

**Supervision:** Runze Huang, Zhangrong Chen.

**Validation:** Zhaoxing Cao.

**Writing – original draft:** Zhaoxing Cao, Fangfang Zhuo.

**Writing – review & editing:** Fei Yan.
